# How narratives influence colorectal cancer screening decision making and uptake: A realist review

**DOI:** 10.1111/hex.12892

**Published:** 2019-04-25

**Authors:** Anke Judith Woudstra, Jeanine Suurmond

**Affiliations:** ^1^ Department of Public Health, Amsterdam Public Health Research Institute, Academic Medical Center University of Amsterdam Amsterdam The Netherlands

**Keywords:** colorectal cancer screening, decision making, Narratives, screening uptake, storytelling

## Abstract

**Background:**

Although narratives have been found to affect decisions about preventive behaviours, including participation in cancer screening, the underlying mechanisms of narratives remain unclear.

**Objective:**

The purpose of this study was to summarize and synthesize existing literature on narrative interventions in the context of colorectal cancer screening. Our main research question was as follows: How, when and for whom do narratives work context of decision making about colorectal cancer screening participation?

**Methods:**

We undertook a realist review to collect evidence on narratives in the context of colorectal cancer screening. A search of the literature was performed in Embase, MEDLINE/PubMed, Cinahl and PsycINFO. We included empirical evaluations (qualitative or quantitative) of narrative interventions. In total, 15 studies met the inclusion criteria. A content‐based taxonomy of patient narrative types in decision aids formed the basis for our initial programme theory.

**Main result:**

We identified four mechanisms: (a) process narratives that address perceived barriers towards screening lead to improved affective forecasting, (b) experience narratives that demonstrate the screening procedure lead to increased self‐efficacy, (c) experience narratives that depict experiences from similar others lead to more engagement and (d) outcome narratives that focus on outcomes of colorectal cancer (CRC) screening decision decrease or increase fear of colorectal cancer. The evidence was limited on which narrative type may facilitate or bias informed decision making in colorectal cancer screening.

**Discussion and conclusion:**

The findings indicate the importance of more detailed descriptions of narrative interventions in order to understand how mechanisms may facilitate or bias informed decision making in colorectal cancer screening.

## 1 INTRODUCTION

Colorectal cancer (CRC) is one of the most common causes of cancer‐related deaths worldwide. Population‐based CRC screening is an effective preventive strategy that significantly reduces CRC morbidity and mortality in the population. However, as with any screening programme, CRC screening has inherent disadvantageous side‐effects (eg, false positives and false negatives) and potential harms associated with colonoscopy. Informed decision making (IDM) about participation in cancer screening has therefore become an explicit purpose of cancer screening programmes in many European countries.^1^ IDM assumes that individuals make a rational and autonomous choice that is based on relevant knowledge and is consistent with their attitude towards undergoing screening.^2^

Previous studies showed that certain groups, including those with lower socio‐economic status (SES), ethnic minority groups and those with lower health literacy levels, are less likely to participate in CRC screening.^3^, ^4^, ^5^ Numerous reasons for lower screening participation in those groups have been suggested, such as lower engagement with cancer screening information, lack of time, financial resources and lower perceived self‐efficacy.^6^ Lately, several studies found that screening invitations, which are typically written materials, are often too difficult to understand and do not help to make informed decisions about health.^7^, ^8^

IDM requires more than just the ability to read and understand cancer screening information. It also requires the ability to appraise the potential benefits and harms of screening and apply the information to one's personal situation.^9^ This combination of skills is referred to as health literacy, which is broadly defined as an individual's capacity to assess, understand and use information to make informed decisions in health care.^10^, ^11^ Poor health literacy is more common among patients who have lower educational level, older patients and racial and ethnic minorities.^12^ In order to reduce inequalities in cancer screening, it is crucial to investigate new communication strategies that are culturally sensitive and are presented in an accessible and comprehensible format.^13^

The inclusion of narratives in cancer screening information is increasingly being suggested as a valuable tool for greater engagement with screening information for ethnic minority groups and people with low SES.^14^ Narratives are personal stories that convey information through others’ health situations or experiences.^14^ Previous research showed that cancer narratives may be especially useful to overcome resistance to cancer screening information and to facilitate the mental simulation of unknown or frightening procedures, including screening.^15^

However, research on narratives interventions and decision making in the context of cancer screening is still in its early stages. It has remained unclear how narratives affect the decision‐making process and cancer screening uptake, and subsequently how they should be used in informed decision making.^16^ Despite their potential to reduce ethnic and socio‐economic inequalities in participation in cancer screening,^14^ narratives can be considered as complex interventions (ie, interventions whose effects are crucially dependent on context and implementation) that may either facilitate or hinder informed decision making in health care.

Narrative interventions work only if they are targeted for participants in the appropriate circumstances and are implemented in the right way. They are also strongly related to the decisions and actions taken by the participants. From a realist perspective, a certain type of narrative may work well in a certain setting but poorly or not at all in another setting.^17^ This knowledge is crucial in order to understand how, when and for whom narratives may work in informed decision making about CRC screening.^17^ A realist approach is a theory‐driven way of analysing complex interventions and is based on the idea that an intervention works (or does not work) because participants make certain decisions or act in a certain way in response to the intervention.^17^

Our main research question was as follows: How, when and for whom do narratives work in the context of decision making about colorectal cancer screening participation?

## 2 METHODS

### 2.1 Design

Following the realist review principles, our methods included: (a) formulating an initial programme theory about how narratives are meant to work and what impacts they are expected to have, (b) selecting and appraising studies and (c) testing the programme theory by extracting, analysing and synthesizing relevant data.^18^ We used the RAMESES publication standards for realist reviews.^19^

### 2.2 Phase I: Initial programme theory

In the first phase, we formulated an initial programme about how a narrative intervention is expected to lead to its effects and in which context it should do so.^17^ One way to develop an initial programme theory is to use concepts from another theory that informs current or comparable interventions.^20^ In addition, countervailing mechanisms can be distinguished (ie, mechanisms that explain why an intervention does not work). We chose the taxonomy by Shaffer and Zikmund‐Fisher^21^ of narratives in decision aids as our initial programme theory. Shaffer and Zikmund‐Fisher^21^ argued that narratives are too often perceived as a homogenous entity and one single construct in existing research. The use of narratives to support decision making should therefore not be classified as “good” or “bad” but rather “whether certain narrative types are suited for certain purposes.” They therefore designed a general taxonomy of narrative content types (ie, “process,” “experience” and “outcome” narratives) in decision aids to better understand when and how narratives affect decisions about health care. These narrative types are hypothesized to depend on three narrative dimensions: purpose, content and overall tone.^21^

### 2.3 Process, experience and outcome narratives

In the context of CRC screening, “process narratives” describe how an individual made the CRC screening decision. “Experience narratives” describe experiential aspects of CRC or CRC screening and essentially provide information about what it is like to have CRC or undergo CRC screening. “Outcome narratives” describe the psychological (eg, patient's quality of life or regret) or physical (eg, CRC patient's survival) health outcomes associated with the CRC screening decision.^21^ The taxonomy hypothesizes that these three narratives types have five different, yet overlapping purposes: (a) to inform, (b) to engage, (c) to model behaviour, (d) to persuade and (e) to comfort. The essential difference between these three narrative types is to the purpose *to inform* (ie, “process” and “experience” narratives) and the purpose *to persuade* (ie, “outcome” narratives). Accordingly, Shaffer and Zikmund‐Fisher^21^ suggest not to use “outcome” narratives as patient decision aids. In contrast, they hypothesize that “experience” narratives as well as “process” narratives might be a helpful component in patient decision aids.

In line with the initial programme theory, we therefore hypothesize that “process” and “experience” narratives may facilitate IDM in CRC screening, whereas “outcome” narratives may be more persuasive and should not be used as decision aids for CRC screening.

### 2.4 Phase II: Selecting and appraising

**Figure 1:**
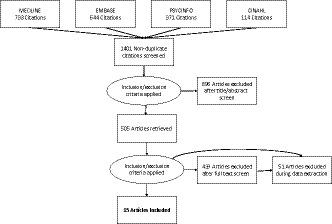
Flow chart of included studies

A literature search for empirical research studies was undertaken to understand, specify and refine the mechanisms of narratives in the context of CRC screening. A clinical librarian (FvE) helped form a search strategy to obtain articles on narrative interventions in the context of CRC screening. See Figure 1 and Appendix S3 for our search strategy. Databases searched included EMBASE, MEDLINE, Cinahl and PsycINFO. We searched for English language articles. Words used were colorectal cancer screening, narrative interventions and health promotion.

Two searches were conducted: the first on 28 December 2016 and an update using the same search strategy on 29 August 2017. Following the realist review principles,^18^ research design was not an exclusion criterion. The inclusion of diverse research designs provided the opportunity to examine the emerging base of evidence on narratives in the context of CRC screening. An included narrative could be an independent intervention but could also be part of a decision aid. We included evaluations (qualitative or quantitative) of narrative interventions in the context of CRC screening. The search resulted in 1401 articles. After checking for duplicates, two authors (AW and JS) screened titles and abstracts. After inclusion and exclusion criteria were applied (see Box 1), 15 studies were accepted for analysis (see Table 1 for the characteristics of the included studies and the narrative interventions).

**Table 1:** Characteristics of included articles and their narrative interventions

Article, setting	Participants	Narrative design	Narrative content type	Main narrative purpose	Narrative content	Overall tone of narrative	Main outcomes
Bennett 2015, UK	19 white men and women (aged 45‐59 y)	First‐person story, Paper‐based	Experience (with outcome components)	Engage (To engage more people in bowel cancer screening, rather than aiding informed decision making)	Four people's experiences of doing the screening test.	Positive	Reduced psychological distress/reduced anxiety Engagement/transportation
Braun 2005, USA	121 Native Hawaiians (aged 50 and older)	First‐person story, presentation	Experience (with outcome components)	Model screening behaviour (To increase self‐efficacy, to model desired behaviour)	A Native Hawaiian CRC survivor told his personal story	Positive (benefits of CRC screening and positive feelings associated with self‐care and survivorship)	No difference in CRC knowledge, attitude, intent and self‐efficacy for narrative vs. non‐narrative delivered by non‐Hawaiian nurse. Ceiling effect of intention suggests inclusion of healthy participants
Cronan 2011, USA	164 Caucasians, African Americans and Mexican Americans living in low‐income neighbourhoods	First‐person story, Videotape	Outcome	Persuade (To test the effectiveness of persuasive messages framed either in gain or loss)	A physician (actor) discussed CRC and the importance of screening.	Positive (benefits of screening) and negative (disadvantages of nog being screened and not detecting cancer early) framed message	Screening uptake increased among Caucasians and Mexican Americans. Message framing made no difference for African Americans and Mexican Americans.
Cueva 2012, USA	172 Alaska Native and American Indian Community Health Workers	Theatre script	Experience (with outcome elements)	Model screening behaviour, Increase comfort (to model ways to talk about CRC screening, increase comfort with talking about CRC)	The characters include Isaac, a man in his early 50s whose father died of colon cancer.	Positive (benefits of screening)	Increased comfort talking to family and friends. Altered behavioural intentions.
Cueva 2013, USA	305 Alaska Native people	Movie script	Experience (with outcome elements)	Model screening behaviour, Increase comfort (To model ways to talk about CRC screening, increase comfort with talking about CRC)	Family and friends discussing screening.	Positive: (benefits of screening)	Increased knowledge Increased comfort Increased shared decision making Altered behavioural intentions Increased screening uptake
Dillard, 2010, USA	1533 predominantly white participants (aged 49‐60 y old)	First‐person story, Paper‐based	Process (with experiential elements)	Inform	Narrative of an individual who had recently made the screening decision.	Positive: (benefits of screening)	Improved affective forecasting Increased risk perception Null effect on knowledge
Dillard 2013, USA	1266 predominantly white participants (aged 49‐60 y old)	First‐ person story, Paper‐based	Process (with experiential elements)	Inform (to educate participants about CRC and screening)	Narrative of an individual who had recently made the screening decision	Positive (benefits of screening) and negative (risks of screening)	Vividness was significantly positively associated with knowledge and behavioural intentions following the narratives, whereas identification was not associated with knowledge
Hwang 2013, USA	153 members of an online weight‐loss community who were not up‐to date with CRC screening (predominantly white women)	First‐person story, online texts	Outcome (with experiential elements)	Model screening behaviour (To connect unscreened individuals to positive role models)	Narratives of members of the same online community	Positive: Benefits of screening	Greater increase screening intention and response efficacy in the narrative group compared to the educational group. No difference in screening uptake between the two groups
Jensen 2014, USA	209 adults (aged 50‐75 y old)	First‐person story	Outcome	Persuade	Tailored and non‐tailored narrative	Negative (Regret of not being screened earlier)	Participants in narrative group were more likely to participate in screening compared to other groups. Narrative interventions were more effective than tailoring at increased screening uptake. Yet, tailoring might be effective for those with high cancer information overload
Larkey, 2007, USA	64 Latinos (aged 18‐85 y old and 86% female)	First‐person story	Experience	Model behaviour (To help learn more about risk for cancer, what one might to do reduce risk for cancer)	Storyline included two sisters that concerned about their father who was scheduled to receive a colonoscopy	Positive (benefits of CRC)	In both interventions, fear and risk perception were decreased somewhat after the intervention
Lipkus 2003, USA	119 men and women (aged 50 y and older)	First‐person story	Outcome	Engage	Perceived severity information included two testimonials from a male and female patient being treated for advanced colon cancer	Negative (manipulate perceptions of CRC risks and severity)	Risk perception Altered behavioural intentions
McGregor, 2015, UK	1221 predominantly White British adults (aged 45‐95,5 y old)	Paper‐based, first‐person story	Outcome (with experiential elements) (the selected quotes and stories predominantly focused on the psychological and physical outcomes to take part in screening)	Model behaviour (To reduce barriers to uptake)	20 (12 females, 8 males) were interviewed about their CRC screening experience	Positive (the overall tone of the leaflet was positive as a consequence of the overwhelmingly positive narratives provided)	Self‐efficacy Reduced psychological distress/reduced anxiety Altered behavioural intentions
McGregor, 2016, UK	150,417 predominantly White British adults (aged 59‐74 y)	Paper‐based, first‐person story	Outcome (with experiential elements) (the selected quotes and stories predominantly focused on the psychological and physical outcomes to take part in screening)	Model behaviour (To reduce barriers to uptake)	20 volunteers (12 females, 8 males) were interviewed about their CRC screening experience	Positive	Screening uptake
Pignone, 2000, USA	249 adults (aged 50‐75 y old)	First‐person story, video	Outcome (with experiential and process elements)	Persuade (Decision screening tests)	Video included information about susceptibility to colon cancer and availability of effective screening tests.	Positive (benefits of CRC screening) and negative (severity of cancer)	Altered behavioural intentions Screening uptake
Shokar, 2006, USA	784 subjects (467 in intervention group, 317 controls). Uninsured predominantly Hispanic individuals	First‐person story, video	Experience (the importance of screening, an explanation of the different tests)	Model (To address barriers, myths, and misconceptions about CRC screening)	Video included information about epidemiology of CRC, the importance of CRC screening and an explanation of the different tests.	Positive (benefits of CRC screening)	Screening uptake

Box 1 **Inclusion and Exclusion criteria**

1 Inclusion criteria

Empirical studies examining the effects of narratives in the context of CRC screening.

Exclusion criteria.

Studies reporting on cancer diagnosis or treatment (and not screening).
Studies describing narratives that solely promote cancer preventive behaviours, such as smoking, nutrition and physical activity, rather than screening.
Studies reporting on the feasibility of narrative interventions in cancer screening. That is, studies that simply examined whether it was possible to use narrative interventions, rather than examine the effects of narratives themselves.

### 2.5 Phase III: Testing the programme theory

To test the programme theory, the included studies were qualitatively analysed, using framework analysis.^22^ Framework analysis is well‐suited for a realist approach, because it can be applied to studies using a mixed‐method design.^22^ Following the framework analysis’ five steps, we first familiarized with the data, using a deductive approach to categorize the included narratives into a narrative content type (ie, “process,” “experience” and “outcome”) according to the initial programme theory. Initially, the authors (AW and JS) reviewed ten articles. In the second step, we used an inductive approach, identifying a thematic framework of mechanisms. The third step (indexing) involved coding the contexts, mechanisms and outcomes in all included studies, for which we used the qualitative data software MAXQDA.^23^ These contexts, mechanisms and outcomes were then compared with the initial programme theory, which we modified using the evaluation findings.

### 2.6 Assessing the quality

Two authors (AW and JS) independently appraised the evidence and generally agreed on the quality of the included articles. Following the realist principles, the assessment of the quality of included studies was guided by Pawsons’^18^ stages of *relevance* and *rigour* (see Appendices S1and S2 )*.* Relevance entails whether the included study can contribute to theory building and rigour entails whether the method used to generate the data is credible and trustworthy. For assessing *relevance,* we focused especially on the theory building in the studies, which can be examined by the use of “thick description” in an article. Lincoln and Guba^24^ describe “thick description” as a way to achieve external validity. Thick description is contrasted with thin description which can be seen as a superficial account (see Box 2). For example, when studies described theoretical concepts in sufficient depth to be relevant to our research questions, we evaluated these as being relevant to our study (see Appendix S1).

Box 2 **Criteria used for assessing relevance**

1

**Table:** 

Thin description	Thick description
Insufficient information to enable the programme theory to be affirmed or replenished	Theoretical concepts are described in sufficient depth to be useful
Largely atheoretical description of narrative intervention	Explanation of theories used
Limited or no consideration of context in which narrative intervention took place	Consideration of context in which narrative intervention took place
Limited or no discussion of the limitations of the methods	Discussion of the limitations of the methods
Description of factors or mechanisms mentioning only ‘an association’ between variables	Description of factors or mechanisms mentioning “model,” “process,” “functions,” “investigates,” “describes,” “explains,” “experiences” etc

For assessing *rigour*, we examined the study design, the data collection and the analysis (see Appendix S2). Disagreements, for example about whether a narrative intervention should be categorized as “outcome” or “experience,” or both, were discussed by both authors (AW and JS) until agreement was reached.

## 3 RESULTS

### 3.1 Overview of included studies

Of the 15 included studies, one study used a qualitative design,^25^ two studies used a mixed methods design,^26^, ^27^ and twelve studies used a quantitative design. Of these twelve studies, nine used a randomized (controlled) trial design. In these randomized (controlled) trial studies, the main intervention was narrative information with the control groups receiving: a culturally targeted presentation,^28^ general information about CRC,^30^, ^32^ a stock (ie, no tailoring, no narrative), tailored narrative or tailored educational message,^33^ a numeric risk tool,^34^ risk information,^35^ an educational video about car safety^36^ and a promotora and video intervention.^37^ Narrative interventions were targeted at various ethnic groups. Three studies were set in the UK, and all other twelve studies were set in the United States (see Table 1).

### 3.2 Narrative content type

By analysing the content of the narratives, we identified only two “process narratives” that informed participants about the decision‐making process of CRC screening.^32^, ^38^ We identified six “experience” narratives that focused primarily on experiences with CRC or with CRC screening. We identified six “outcome” narratives, described by seven studies 30, 33, 35, 36, 39 that focused primarily on the outcomes of the CRC screening decision (see Table 1).

### 3.3 Mechanisms of narrative content types

**Figure 2:**
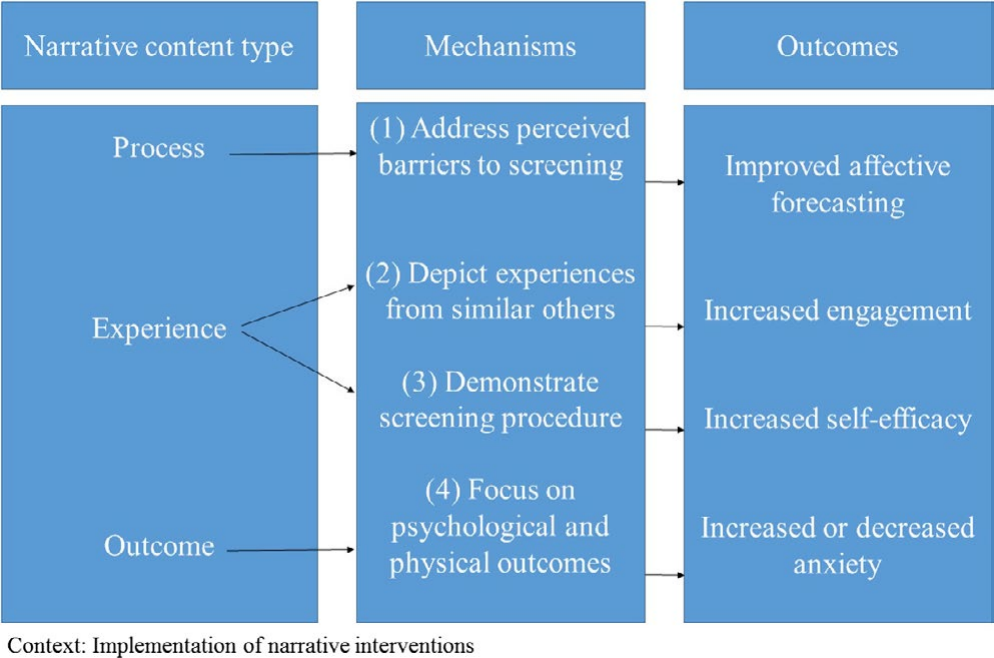
Final programme theory

From the framework analysis, we identified four mechanisms and eight associated countervailing mechanisms (see Figure 2 for the final programme theory).

#### 3.3.1 Mechanism 1 Process narratives that address perceived barriers towards screening lead to improved affective forecasting

The process narratives^32^, ^38^ that we identified described an experience from a similar other who had recently made the screening decision. In these narratives, the character first felt uncertain about screening and knew little about screening tests. The character then talked about the decision process, including encountered barriers towards screening (eg, inconvenience of the screening procedure including having to take the laxative). Dillard and Fagerlin^32^ described how the narrative intervention was tailored to participants’ overestimated barriers towards CRC screening, so‐called “affective forecasts.” For instance, the narrative character stated: “When trying to decide about a colonoscopy, I spent a lot of time thinking about what the experience would be like.” Dillard and Main^38^ reported how the narrative information also encouraged participants to think about the potential harms and benefits of screening and talking to a physician, in order to make a deliberate decision. By addressing affective forecasting errors, this narrative intervention reduced perceived barriers to CRC screening. Addressing perceived barriers towards screening was shown to increase participants’ interest towards screening, reduce perceived barriers and improve affective forecasting. This mechanism may work especially for individuals who overestimate barriers towards screening. This narrative intervention, however, had the same effect on knowledge compared to the educational intervention, as both interventions contained personally relevant CRC information for all participants eligible for screening.^32^

***Countervailing mechanism 1.1:*** This mechanism may not be effective for underserved populations if narratives do not address system barriers, such as transportation and costs.

#### 3.3.2 Mechanism 2 Experience narratives that demonstrate the screening procedure lead to increased self‐efficacy

Several “experience” narratives demonstrated the screening procedure. For example, in the study by Braun and Fong,^28^ a native Hawaiian physician provided instructions on testing and demonstrated how to use the FOBT kit to collect stool samples using a child's potty and Play‐Doh stool.^28^ If narratives demonstrated how to do the test,^25^, ^28^, ^30^, ^31^ then the narrative audience felt more confident that they could do the test themselves and this helped them to make a decision about the screening. This in turn led to increased self‐efficacy in relation to test completion^25^, ^28^, ^30^ Another study^29^ also suggested that self‐efficacy increased because participants read about the experiences from individuals who had already undergone colorectal cancer screening. In the study by Bennett and von Wagner,^25^ participants felt that the narrative information provided reassurance about the perceived unpleasantness of the screening test: “It takes some of the yuck factor away in that you think, well these people are just ordinary people and they've been through it.”

***Countervailing mechanism 2.1:*** This mechanism might not be effective if underserved participants are not provided with free or low cost home screening test kits, or when the narrative information is not being implemented simultaneously with the screening test kit. 31

#### 3.3.3 Mechanism 3 Experience narratives that depict experiences from similar others lead to more engagement

Several “experience” narratives presented CRC or CRC screening experiences from others who were depicted as “just ordinary people” from the community,^25^ as “real” people,^31^ similar others,^32^ but also as respected role models in the community.^28^ For instance, one participant in the study by Bennett and von Wagner^25^ stated: “saying what they do and that they're married and they have children make you identify a little bit more with them.” These “experience” narratives showed that if narratives present a range of experiences from similar others, this makes narrative information more reassuring, more credible, more vivid, more engaging,^32^ helps to reduce fears people have about the test^38^ and legitimizes the quotes and stories.^30^ This leads to normalization of cancer screening,^25^, ^28^ increased knowledge about CRC, and an increase in intentions to seek more information about screening.^38^ Experience narratives modelled CRC screening conversations in a variety of social settings. By integrating humour within the narrative information, resistance towards cancer messages was reduced and comfort with talking about screening was increased. The participants watching the narrative reported that they felt more comfortable talking about cancer screening because they could listen to the words the characters used.^26^

***Countervailing mechanism 3.1:*** This mechanism may not work if the depicted experiences are perceived as too highly personalized. Highly personalized stories can be viewed as persuasive and manipulative and hence generate resistance to the message^25^, ^33^ rather than stimulate informed decision making about CRC screening. Yet, when a narrative depicts multiple experiences of CRC screening, this enables participants to relate to at least one person's experience, resulting in the narrative being perceived as credible and reliable.^25^
***Countervailing mechanism 3.2:*** This mechanism may work only if both vividness and identification are part of the engagement process. Dillard and Main^38^ found that vividness is more strongly associated with CRC screening knowledge and behavioural intentions than perceived identification. Cueva and Kuhnley^27^ showed that both Caucasian and Alaska native participants could relate with the addressed barriers in the narrative, indicating that the narrative information was perceived as being vivid by both groups.
***Countervailing mechanism 3.3:*** This mechanism may not work among certain ethnic groups if the narrative information does not consider specific cultural values, beliefs and traditions. In most narrative interventions, the target group was involved in the development and evaluation of the narrative. Specifically, the use of familiar lay terms, availability in different languages, recruiting actors from the community and the setting of a typical home or clinic, can increase perceived similarity with the narrative character.^26^, ^27^, ^34^
***Countervailing mechanism 3.4:*** When solely presenting narrative information, this mechanism might not be effective for those who also regard factual information as essential to making a decision about CRC screening.^25^

#### 3.3.4 Mechanism 4 Outcome narratives that focus on outcomes of the CRC screening decision increase or decrease anxiety

There was a wide variety in the framing of the psychological and physical outcomes of CRC screening within the identified “outcome” narratives. Whereas the overall tone within most narrative interventions was positive (eg, feeling lucky to have had cancer picked up early), other narratives were more negatively framed (eg, reporting individual's regret of not getting screened earlier). For example, in the narrative described by Jensen and King,^33^ the character, who was diagnosed with colorectal cancer, stated: “I should have been screened earlier. I knew it was important. I was taking care of everything but myself.” Lipkus and Green^35^ found that negatively framed messages (ie, about the severity of CRC) were more effective in increasing screening intentions of individuals compared to those who did not receive severity information. Negatively framed cancer messages generated fear of cancer by presenting experiences from patients living with CRC who emphasized how the disease negatively affected their lives. However, stronger screening intentions among those who received severity information were generally not maintained at the 6‐month follow‐up.

On the contrary, Cronan and Conway^39^ found that positively framed messages (eg, about the benefits of screening participation) were more effective in increasing screening uptake than negatively framed messages (eg, disadvantages of not being screened earlier and not detecting cancer early) among Caucasians. The study by Larkey and Gonzalez^34^ did not find any effect of a narrative that included information about the risk for CRC and the benefits of CRC screening (“if a polyp is found and removed, CRC may be prevented”) on anxiety or fear for CRC.

***Countervailing mechanism 4.1:*** Message framing (either positive or negative) may not work for certain groups, if the narrative is not tailored to personal characteristics. For example, Cronan and Conway^39^ found that the message framing did not work for African Americans and Mexican Americans in their study, as the message was not culturally tailored and consequently might therefore not have been perceived as relevant for all ethnic groups. If narratives included oral storytelling traditions, values of humour and values of family and community, this made narratives more culturally appropriate and appreciated by different ethnic groups.^27^
***Countervailing mechanism 4.2:*** This mechanism may not facilitate informed decision making, as the focus on psychological and physical outcomes of cancer screening, either positively or negatively framed, can be selective and misleading.^33^ For instance, certain narratives may only present unusual cancer screening events and focus on the benefits of cancer screening only, which may make cancer screening seem normative.

### 3.4 Mode of information

Narrative information might be effective in influencing decision making, regardless of the mode of information (paper‐based, video, promotora‐only).^37^ In addition, Pignone and Harris^36^ found that narrative information, in combination with an educational brochure that is tailored to an individual's decision‐making stage, increased screening intention and uptake. Studies focusing on diverse groups showed that as long as the presenter of the narrative information is culturally sensitive, the narrative can be successfully presented or delivered by any individual, regardless of ethnic background.^28^, ^37^ However, the narrative information must be easily accessible to the participants. Hwang and Ottenbacher,^29^ for instance, found that online narrative information was only accessed by fewer than 60% of the participants. This low participation rate may have resulted from the requirement of joining an online team. Additionally, McGregor and von Wagner^30^ showed that narrative information might increase information overload when it is being added to existing CRC screening information.

## 4 DISCUSSION

The aim of this review was to understand how, when and for whom narratives work in the context of CRC screening. Using the taxonomy by Shaffer and Zikmund‐Fisher^21^ as our initial programme theory, we categorized the included narrative interventions into three narrative types: “process,” “experience” and “outcome.” We identified only two “process” narrative interventions that targeted the decision‐making process, six “experience” narratives and six “outcome” narratives. We further specified and refined four mechanisms, which provide an exploratory account of how these narrative content types may work in decision making about CRC screening. The following four mechanisms were identified: (a) process narratives that address perceived barriers towards screening lead to improved affective forecasting, (b) experience narratives that demonstrate the screening procedure lead to increased self‐efficacy, (c) experience narratives that depict experiences from similar others lead to more engagement and (4) outcome narratives that focus on outcomes of the CRC screening decision decrease or increase fear of CRC.

With regard to countervailing mechanisms, we found that CRC screening narrative interventions may not work when system barriers (eg, costs or transportation) are not addressed, when free test kits are not provided, when the narrative information is not implemented simultaneously with the screening test kit, when the narrative is perceived as too highly personalized, as not being vivid or as not being culturally relevant. This implies that in the development of narrative interventions, public health practitioners should first try to understand how specific values and beliefs about CRC and CRC screening are important to the target group. This requires tailoring cultural characteristics at the individual level, and not just at the group level (ie, cultural targeting).^40^

When implementing narrative interventions into practice, attention should be given to the length of the narrative information and the appropriate timing of the narrative information. Implementing CRC screening at the pre‐invitation stage might not increase the engagement of participants at that certain time point. 31 With regard to “experience” narratives and “process” narratives, certain mechanisms might lead to outcomes (eg, self‐efficacy or affective forecasting) that might facilitate IDM about CRC screening. However, most narrative interventions included elements of multiple narrative types. It is therefore impossible to disentangle whether mechanisms of certain narrative types truly facilitate or bias informed decision making. With regard to “outcome” narratives, our findings showed that public health practitioners should be cautious when emphasizing the benefits of CRC screening only, as this is selective and misleading in decision making about CRC screening. In addition, positively framed messages might be too reassuring, whereas negatively framed messages might increase fear and bias risk perception. In line with Shaffer and Zikmund‐Fisher,^21^ we suggest that “outcome” narratives should not be used as decision aids. Yet, our findings suggest that all narrative types potentially bias decision making as most of them presented one‐sided information about the potential benefits of screening. To conclude, there is too little evidence to recommend which mechanisms of narrative types can be employed in the context of IDM about CRC screening.

CRC screening programmes must ensure that screening invitees receive accurate information based on the most recent available evidence and information about the potential benefits and harms of CRC screening.^41^ Accordingly, decision aids should inform about these benefits and harms of screening, and should present these in terms of absolute risk, not relative risk. Moreover, the information must not be directive and must facilitate an autonomous choice.^42^

### 4.1 Limitations

This synthesis of literature on narrative interventions in the context of cancer screening has some important limitations. First, the narrative interventions varied in terms of narrative length, intervention (eg, video, leaflet, and testimonial), purpose, content, overall tone and participants, making it difficult to compare between the studies and categorize the narrative interventions. Second, few narratives have been experimentally evaluated and the majority of quantitative studies used a cross‐sectional design, precluding causal inference. Third, none of the studies assessed decisional certainty, deliberation or informed decision making. Therefore, we are not certain whether the narrative interventions facilitated decision making that was congruent with participants’ values and preferences. Fourth, it might be possible that we missed studies on CRC screening narratives as we focused on studies evaluating narrative effects. Studies that solely described the development of narrative, for example, were excluded. Fifth, most narrative interventions in the studies were carried out in the United States, which means that the development process of narrative interventions reflects the access to health insurance and care provided in public and private systems. In addition, the focus in the United States is more on screening uptake rather than on informed decision making, through actively promoting the message “the best test is the one that gets done.”^43^ Hence, our synthesis provided more empirical evidence on the positive impact of narrative interventions in promoting screening uptake than on its influence on informed decision making.

## 5 CONCLUSION

An important finding of this review is that narratives that solely describe the decision‐making process are hugely underrepresented in the literature on CRC screening narratives. Future studies must report on the purpose and the content of narrative interventions more clearly in order to understand which and how narrative types lead to which outcomes and for whom they work (and do not work) in the context of decision making about CRC screening participation.
