# Supplementary material for: How narratives influence colorectal cancer screening decision making and uptake: A realist review
Source: Health Expect. 2019 Apr 25;22(3):327–37. doi: 10.1111/hex.12892 (PMC6543268; doi:10.1111/hex.12892)
Supplement: Supplementary file 1 [file HEX-22-327-s001.docx]

**Appendix 1: Assessment of relevance**

|  | **Theoretical concepts are described in sufficient depth to be useful** | **Explanation of theories used** | **Consideration of context in which narrative intervention took place** | **Discussion of the limitations of the methods** | **Description of factors or mechanisms** |
| --- | --- | --- | --- | --- | --- |
| Bennett 2015 | Thick | Thick | Thick | Thick | Thick |
| Braun 2005 | Thick | Thick | Thick | Thick | Thick |
| Cronan 2011 | Thick | Thick | Thick | Thick | Thin: No explanation for why people from different ethnic backgrounds may process information differently. There was limited power to explore potential moderators of the found effects. |
| Cueva 2012 | Thin: Brief mentioning of principles of Indigenous methodologies | Thin | Thick | Thin: Not described | Thin: Evaluation of the script, but no explanation of effects |
| Cueva 2013 | Thick | Thin: No description in results how theory is used | Thick | Thin: Not described | Thin: Evaluation of the movie but no explanation of effects |
| Dillard, 2010 | Thick | Thick | Thick | Thick | Thick |
| Dillard 2013 | Thick | Thick | Thick | Thick | Thick |
| Hwang 2013 | Thin: Brief mentioning of Social norms | Thin | Thick | Thick | Thin: No explanation of effects |
| Jensen 2014 | Thick | Thick | Thick | Thick | Thin: Mechanism of effect is unclear. Ten mediator variables were examined and none of them explained the relationship between exposure to narrative and CRC screening behaviour |
| Larkey, 2007 | Thick | Thick | Thick | Thin: No clear description | Thin: Lack of demonstration of how mediating factors explain mechanisms |
| Lipkus, 2003 | Thick | Thick | Thick | Thick | Thick |
| McGregor, 2015 | Thick | Thick | Thick | Thick | Thick |
| McGregor, 2016 | Thin: No description of theories | Thin: No explanation of theories | Thick | Thick | Thin: Focus on implementation of narrative and timepoints but not on underlying mechanisms |
| Pignone, 2000 | Thin: Minor description of theory used | Thin: No explanation of theories | Thick | Thin: No clear description | Thin: Lack of demonstration of how mediating factors explain narratives |
| Shokar, 2016 | Thick | Thick | Thick | Thick | Thin: No explanation of mechanisms |

**Appendix 2: Assessment of rigor (study design, data collection and data analysis)**

|  | How rigorous is the research design  *For example:*  - Is the design appropriate to the research question?  - Are there clear accounts of the rationale/justification for the sampling, data collection and data analysis techniques used?  - Is the selection of cases/sampling strategy theoretically justified? | How well was the data collection carried out?  *For example:*  - Are the data collection methods clearly described?  - Were the appropriate data collected to address the research question? | Is the data analysis sufficiently rigorous?  *For example:*  - Is the procedure explicit – is it clear how the data were analyzed to arrive at the results?  - Is it clear how the data and themes and concepts were derived from the data? |
| --- | --- | --- | --- |
| Bennett 2015 | + | + | + |
| Braun 2005 | + | + | + |
| Cronan 2011 | - Relatively small number of participants within the subgroups | + | + |
| Cueva 2012 | - | + | - No description of data analysis method |
| Cueva 2013 | - | + | - No description of data analysis method |
| Dillard, 2010 | + | + | + |
| Dillard 2013 | + | + | + |
| Hwang 2013 | + | + | + |
| Jensen 2014 | + | + | + |
| Larkey, 2007 | - Pilot study with small sample: some of the results are inconclusive | + | + |
| Lipkus 2003 | - Pilot study with small sample | + | + |
| McGregor, 2015 | - Variation in socioeconomic deprivation was minimal in both groups. People who decided not to take part may not have been reached. | + | + |
| McGregor, 2016 | + | + | + |
| Pignone, 2000 | + | + | + |
| Shokar, 2016 | + | + | + |

Appendix 3: Search Strategy

**MEDLINE (Ovid):**

Database(s): Ovid MEDLINE(R) Epub Ahead of Print, In-Process & Other Non-Indexed Citations, Ovid MEDLINE(R) Daily and Ovid MEDLINE(R) 1946 to Present 
Search Strategy:

| **#** | **Searches** | **Results** |
| --- | --- | --- |
| 1 | *Colorectal Neoplasms/ or *Breast Neoplasms/ or *Uterine Cervical Neoplasms/ or (breast cancer or cervical cancer or colorectal cancer).ti,ab,kw. or cancer.ab. | 1741999 |
| 2 | Mass Screening/ or Mammography/ or Neoplasms/di or exp Breast Neoplasms/di or Uterine Cervical Neoplasms/di or exp Colorectal Neoplasms/di or (cancer screening or mass screening or mammograph*).ti,ab,kw. | 176726 |
| 3 | health education/ or Health Promotion/mt or patient education as topic/ or *Education, Medical/mt or *Health Knowledge, Attitudes, Practice/ or *Attitude to Health/ or (program* or promot* or recommend* or informed choice or informed decision or decisionmaking or decision making).ti,ab,kw. | 2849289 |
| 4 | communication/ or verbal behavior/ or Narration/ or anecdotes as topic/ or *Cultural Characteristics/ or exp Video Recording/ or (video* or movie* or visual arts intervention* or arts-based activit* or DVD intervention* or tailor* or culturally target* or narrat* or story tell* or storytell* or story or stories or cancer control intervention*).ti,ab,kw. or interventions.ti. | 519013 |
| 5 | (education* or public health or social science).af. or narrat*.ti. | 2328489 |
| 6 | (biomarker* or human papillomavirus or cost-benefit analysis or advanced proximal neoplasia or prostate or lung or skin or regression model* or diagnostic test accuracy).af. | 3363703 |
| 7 | (1 and 2 and 3 and 4 and 5) not 6 | 730 |
| 8 | storytelling.ti. and Anecdotes as Topic/ | 43 |
| 9 | 7 or 8 | 773 |

**EMBASE (Ovid):**

Database(s): Embase Classic+Embase 1947 to 2017 August 29 
Search Strategy:

| **#** | **Searches** | **Results** |
| --- | --- | --- |
| 1 | *colorectal cancer/ or *colon cancer/ or (colorectal cancer or colon cancer or bowel cancer).ti,ab,kw. | 176137 |
| 2 | exp verbal communication/ or (narrative or narration or verbal communication or story tell* or storytell* or survivor stor* or educational intervention* or telenovela or testimonial* or video*).ti,ab,kw. | 432909 |
| 3 | *mass screening/ or *cancer screening/ or *early diagnosis/ or *health promotion/ or *attitude to health/ or *health behavior/ or (screening or behavioral intention* or knowledge attitude*).ti,ab,kw. | 726463 |
| 4 | 1 and 2 and 3 | 644 |

**PSYCINFO (Ovid):**

Database(s): PsycINFO 1806 to August Week 3 2017 
Search Strategy:

| # | Searches | Results |
| --- | --- | --- |
| 1 | *Neoplasms/ or (colorectal cancer or colon cancer or bowel cancer).ti,ab,id. | 180248 |
| 2 | narratives/ or storytelling/ or verbal communication/ or exp oral communication/ or (narrative or narration or verbal communication or story tell* or storytell* or survivor stor* or educational intervention* or telenovela or testimonial* or video*).ti,ab,id. | 275697 |
| 3 | *Cancer Screening/ or *Health Behavior/ or *Health Education/ or (screening or behavioral intention* or knowledge attitude*).ti,ab,id. | 661458 |
| 4 | 1 and 2 and 3 | 371 |

**CINAHL (Ebsco)**

| **#** | **Query** | **Results** |
| --- | --- | --- |
| S1 | ( ( (MM "Colorectal Neoplasms") OR ( TI ( (colorectal cancer) ) OR AB ( (colorectal cancer) ) ) ) ) AND ( ( (MH "Narratives") OR (MH "Storytelling") OR ( TI ( (narrative or narration or verbal communication or story tell* or storytell* or survivor stor* or educational intervention* or telenovela or testimonial* or video*) ) OR AB ( (narrative or narration or verbal communication or story tell* or storytell* or survivor stor* or educational intervention* or telenovela or testimonial* or video*) ) ) ) ) AND ( ( ( ((MM "Attitude to Health") ) OR (MH "Health Behavior") OR (MH "Health Education") OR (MH "Health Promotion") OR (MH "Cancer Screening") OR ( TI ( cancer screening or screening or screening or behavioral intention* or knowledge attitude) ) OR AB ( cancer screening or screening or screening or behavioral intention* or knowledge attitude) ) ) ) ) | 114 |
